# Brain proteomic atlas of alcohol use disorder in adult males

**DOI:** 10.1038/s41398-023-02605-0

**Published:** 2023-10-13

**Authors:** Pang-ning Teng, Waleed Barakat, Sophie M. Tran, Zoe M. Tran, Nicholas W. Bateman, Kelly A. Conrads, Katlin N. Wilson, Julie Oliver, Glenn Gist, Brian L. Hood, Ming Zhou, G. Larry Maxwell, Lorenzo Leggio, Thomas P. Conrads, Mary R. Lee

**Affiliations:** 1 Women’s Health Integrated Research Center, Henry M. Jackson Foundation for the Advancement of Military Medicine, Inc., Bethesda, MD USA; 2 Women’s Health Integrated Research Center, Women’s Service Line, Inova Health System, Falls Church, VA USA; 3 Clinical Psychoneuroendocrinology and Neuropsychopharmacology Section, Translational Addiction Medicine Branch, National Institute on Drug Abuse and National Institute on Alcohol Abuse and Alcoholism, National Institutes of Health, Baltimore, Bethesda, Maryland USA; 4 Medication Development Program, National Institute on Drug Abuse Intramural Research Program, National Institutes of Health, Baltimore, Maryland USA; 5 Center for Alcohol and Addiction Studies, Department of Behavioral and Social Sciences, School of Public Health, Brown University, Providence, Rhode Island USA; 6 Division of Addiction Medicine, Department of Medicine, School of Medicine, Johns Hopkins University, Baltimore, Maryland USA; 7 Department of Neuroscience, Georgetown University Medical Center, Washington, DC USA; 8 Veterans Affairs Medical Center, Washington, DC USA

**Keywords:** Addiction, Molecular neuroscience

## Abstract

Alcohol use disorder (AUD) affects transcriptomic, epigenetic and proteomic expression in several organs, including the brain. There has not been a comprehensive analysis of altered protein abundance focusing on the multiple brain regions that undergo neuroadaptations occurring in AUD. We performed a quantitative proteomic analysis using a liquid chromatography-tandem mass spectrometry (LC-MS/MS) analysis of human postmortem tissue from brain regions that play key roles in the development and maintenance of AUD, the amygdala (AMG), hippocampus (HIPP), hypothalamus (HYP), nucleus accumbens (NAc), prefrontal cortex (PFC) and ventral tegmental area (VTA). Brain tissues were from adult males with AUD (*n* = 11) and matched controls (*n* = 16). Across the two groups, there were >6000 proteins quantified with differential protein abundance in AUD compared to controls in each of the six brain regions. The region with the greatest number of differentially expressed proteins was the AMG, followed by the HYP. Pathways associated with differentially expressed proteins between groups (fold change > 1.5 and LIMMA *p* < 0.01) were analyzed by Ingenuity Pathway Analysis (IPA). In the AMG, adrenergic, opioid, oxytocin, GABA receptor and cytokine pathways were among the most enriched. In the HYP, dopaminergic signaling pathways were the most enriched. Proteins with differential abundance in AUD highlight potential therapeutic targets such as oxytocin, CSNK1D (PF-670462), GABA_B_ receptor and opioid receptors and may lead to the identification of other potential targets. These results improve our understanding of the molecular alterations of AUD across brain regions that are associated with the development and maintenance of AUD. Proteomic data from this study is publicly available at www.lmdomics.org/AUDBrainProteomeAtlas/.

## Introduction

Alcohol use disorder (AUD) is a leading cause of mortality and morbidity and a risk factor for many physical and psychiatric disorders [1]. The development and maintenance of AUD are conceptualized as a progression from alcohol binging/intoxication to preoccupation/craving and, finally, negative reinforcement [2]. The neurocircuitry underlying these stages is centered on the basal ganglia, prefrontal cortex (PFC) and extended amygdala (AMG), respectively [2]. In addition, alcohol abuse has been shown to impact the endocrine system, including the hypothalamus (HYP) [3]. Understanding the molecular alterations associated with AUD in these brain regions can lead to the identification of new targets for AUD treatment [4].

Previous studies have compared differentially expressed genes in individuals with AUD with matched controls in some of the brain regions that are involved in the initiation and maintenance of AUD, specifically the PFC, AMG, nucleus accumbens (NAc), hippocampus (HIPP) and ventral tegmental area (VTA) [5]. In these brain regions, RNA-seq and microarray data have identified expression differences in epigenetic and miRNA regulation as well as non-coding RNA, ion channel, signal transduction, immune, stress response and metabolism pathways. However, gene expression is not always an accurate indicator of protein abundance, as the latter is affected by epigenetic and post-transcriptional modifications [6, 7]. Therefore, studying changes in the proteome as a consequence of AUD offers a deeper and more mechanistically relevant understanding of the neurobiology of AUD [4].

There have been several case control studies investigating proteome alterations in the brains of individuals with AUD [8–16]. Proteomic studies of the human brain in individuals with AUD have focused on the PFC as this region is a common site of functional [16] and anatomic [11] alterations in individuals with AUD. In a multi-region proteomic study focusing on subcortical brain regions (caudate nucleus, putamen and NAc), several neurotransmitters (norepinephrine, choline, acetylcholine, histamine, glutathione, GABA, tyrosine, dopamine) were reduced in these brain regions in the AUD group compared to controls [17]. A more recent study used sequential window acquisition of all theoretical mass spectra (SWATH-MS) proteomics and reported alterations in metabolic pathways, including glycolysis, cytoskeleton trafficking, and PFC excitotoxicity as well as the motor cortex of individuals with AUD compared to controls [18].

Building on these studies, which examine a single or limited number of brain regions, we conducted a deep quantitative proteomics analysis of postmortem brains from AUD and matched control individuals from several cortical and subcortical brain regions, including the AMG, PFC (superior frontal Brodmann areas 8 and 9), HIPP, VTA, NAc, and HYP that are known to be involved in neuroadaptations that occur with heavy, compulsive alcohol drinking. Proteomic pathway alterations in each brain region were identified using Ingenuity Pathway Analysis (IPA, Qiagen). These analyses revealed important proteomic pathway alterations unique to AUD individuals and provided insights into potential therapeutic targets (from preclinical or clinical studies) that are constituents in the pathway alterations identified in AUD individuals.

## Materials and methods

### Subjects

Human male postmortem brain tissue samples (fully de-identified) with a current diagnosis of AUD, severe (*n* = 11, diagnostic and statistical manual of mental disorders (DSM-5)) [19], and matched control individuals without AUD (*n* = 16) were obtained from the New South Wales Tissue Resource Centre (NSWBTRC) at the University of Sydney, Australia (Supplementary Table 1) [20]. The project was approved by the National Institute on Alcohol Abuse and Alcoholism (NIAAA) Scientific Advisory Board and exempted from review by the National Institutes of Health (NIH) Institutional Review Board, as determined by the NIH Office of Human Subjects Research Protections. All individuals with AUD were daily drinkers, had alcohol detected in their blood and were daily smokers at the time of death; only one control subject was a daily smoker at the time of death. The method of clinical and behavioral assessments has been previously described [21].

### Tissue specimen preparation

Unstained and deparaffinized formalin-fixed, paraffin-embedded (FFPE) brain tissue sections were imaged using an Aperio ScanScope XT slide scanner (Leica Microsystem, Feasterville, PA). Area measurement of tissue sections was performed using the Aperio eSlide Manager Software (Leica Microsystems). Tissue samples from the six brain regions (AMG, HIPP, HYP, NAc, PFC, VTA) were scraped into 20 µL of 100 mM tetraethylammonium bicarbonate (TEAB), 10% acetonitrile in MicroTubes and capped with MicroCaps (Pressure Biosciences, Inc.) with a maximum tissue area of 200 mm^2^. Pressure-assisted digestion was performed as previously described using SMART Digest Trypsin (2 µL, Thermo Fisher Scientific) and a Barocycler 2320EXT (Pressure BioSciences) [22]. Peptide samples were transferred to 0.5 mL tubes, lyophilized and resuspended in 100 mM TEAB (pH 8.0), and peptide concentration was determined using a bicinchoninic acid assay (Thermo Fisher Scientific). A total of 12 slides were available for most of the cases (20 out of 27 total cases). Three slides (slide 1, 6 and 12) from each sample were selected for scraping to represent the top, middle and bottom of the tissue block. Each tissue scrape was digested individually with trypsin, and the resultant peptides were pooled prior to quantification. For the seven samples that had less or greater than 12 slides available, slides representing the top, middle and bottom of the tissue block were sampled accordingly.

### Tandem mass tag labeling of peptides

Peptides were labeled with tandem-mass tag (TMT) isobaric labels (TMTpro 16plex™ Isobaric Label Reagent Set, Lot UL296296, Thermo Fisher Scientific). Ten micrograms of each sample were aliquoted into a final volume of 100 µL of 100 mM TEAB, and peptides were labeled according to the manufacturer’s protocol. Multiplexed samples were fractionated by high pH reversed-phase chromatography (1260 Infinity II, Agilent Technologies) as previously described [23]. Each multiplex contained a TMT channel of pooled samples specific for a given brain region, a channel of pooled samples representing all the brain regions and 14 channels corresponding to 14 individual patient samples. Twenty-four concatenated fractions were generated for global LC-MS/MS analysis.

### LC-MS/MS and data analysis

Liquid chromatography-tandem mass spectrometry (LC-MS/MS) analyses were performed on a nanoflow high-performance LC system (EASY-nLC 1200, Thermo Fisher Scientific) coupled online with an Orbitrap mass spectrometer (Q Exactive HF-X, Thermo Fisher Scientific) as previously described [23]. Global protein-level abundances were generated from peptide spectral matches identified by searching .raw data files with a publicly available, non-redundant human proteome database (Swiss-Prot, http://www.uniprot.org, downloaded 12-01-2017) using Proteome Discoverer (v2.2.0.388, Thermo Fisher Scientific), Mascot (v2.6.0, Matrix Science), and in-house tools using identical parameters as previously described [22].

### Bioinformatics and statistical analysis

Sample data from technical replicates were excluded from the global proteome or brain regional analyses when Spearman correlation ρ < 0.6. In the case when Spearman correlation ρ ≥ 0.6 for two technical replicates; data from one of the replicates was selected at random for the downstream analyses. Global proteome data was visualized by principal component analysis (PCA) and the top 100 most variable proteins were visualized in a heatmap using Plotly [24]. A group comparison of the global proteome was conducted for each brain region using the linear models for microarray data (LIMMA) package (v3.8) in R (v3.5.2). Protein alterations passing LIMMA *p*-value < 0.01 were assessed and visualized by volcano plot. Total proteins identified from each brain region and proteins passing LIMMA *p*-value < 0.01 and FC cutoff ±1.5 (Log_2_FC = 0.585) were visualized by upset plots.

### Ingenuity Pathway Analysis (IPA)

Differentially expressed proteins between AUD and control groups (LIMMA *p* < 0.01, fold-change (FC) cutoff of ±1.5 (Log_2_FC = 0.585)) were submitted to IPA to evaluate gene ontology, canonical pathways and potential drug targets previously [25]. Cellular compartment and molecular function profiles of these differentially expressed proteins were analyzed for each brain region. Brain region differences in the number of differentially expressed proteins for cellular compartment and molecular function were assessed with Fisher’s exact test. For the canonical pathway analysis, input literature was limited to mammalian neurological tissues (Score cutoff −log(*p*-value) = 1.3 as default). The activation or inhibition states of the canonical pathways were predicted based on a *z*-score algorithm that was calculated based on gene expression patterns and correlation with IPA-curated literature findings.

## Results

### Proteomic characterization by brain region

**Fig. 1:**
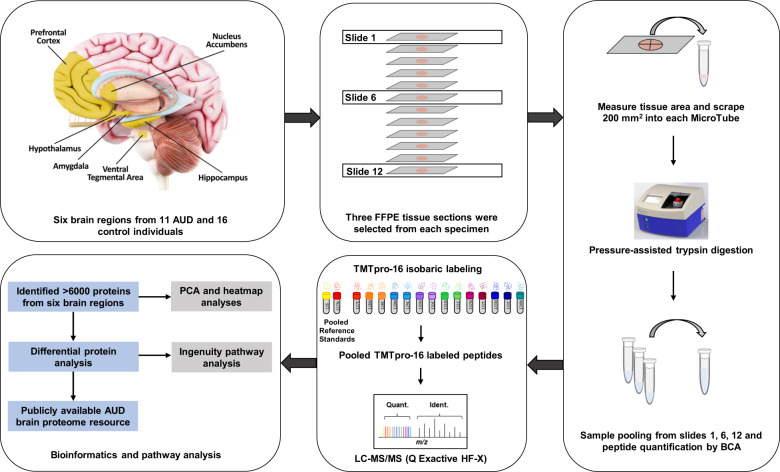
Overview of the experimental workflow. FFPE tissue from six brain regions of adult AUD (*n* = 11) and control (*n* = 16) individuals were harvested for quantitative proteomic analysis.

FFPE tissue from six brain regions of AUD and control adult males were harvested for quantitative proteomic analysis (Fig. 1). The total number of proteins identified from each brain region (AMG, HIPP, HYP, NAc, PFC and VTA) for AUD (*n* = 16) and control (*n* = 11) individuals is shown in Table 1. A total of 6132 proteins were identified from the entire sample set, and 4323 proteins (global brain proteome) were identified in ≥50% of the samples regardless of the brain region (Supplementary Table 2).

**Table 1:** Proteins identified and quantified in each brain region.

Brain region	Total number of proteins quantified	Number of differentially abundant proteins (FC cutoff = 1.5, *p* < 0.01)
**AMG**	4333	87
**HIPP**	4597	14
**HYP**	4541	39
**NAc**	5093	34
**PFC**	4936	19
**VTA**	5137	29

*AMG* amygdala, *HIPP* hippocampus, *HYP* hypothalamus, *NAc* nucleus accumbens, *PFC* prefrontal cortex, *VTA* ventral tegmental area, *FC* fold change.

**Fig. 2:**
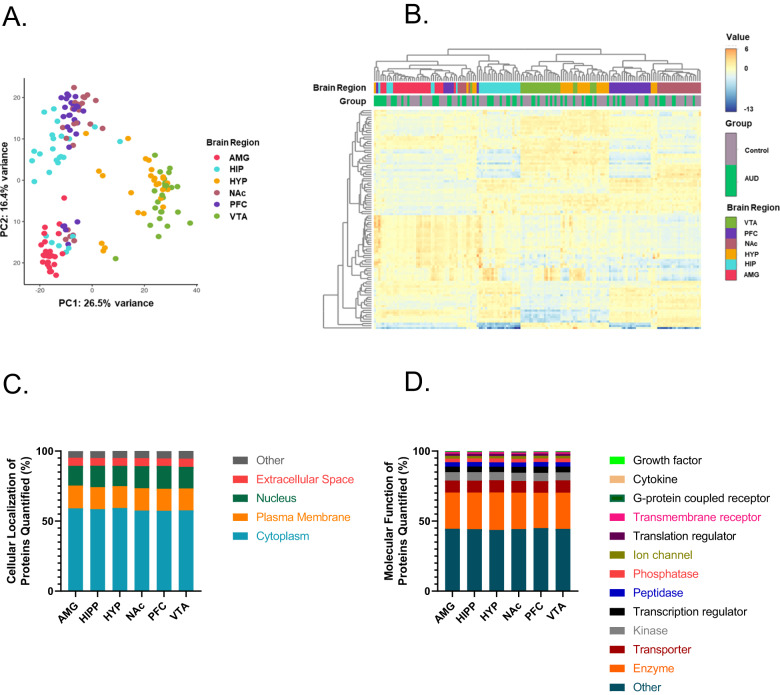
Global brain proteome. **A** PCA of global brain proteomics. **B** Hierarchical clustering of top 100 most variable proteins. **C** Cellular localization of total proteins identified from each brain region. **D** Molecular functions of total proteins identified from each brain region. AMG amygdala, HIPP hippocampus, HYP hypothalamus, NAc nucleus accumbens, PFC prefrontal cortex, VTA ventral tegmental area.

Principle component analysis (PCA) of the global brain proteome demonstrated strong clustering by brain region (Fig. 2A). A similar pattern of clustering by brain region rather than the subject group was observed in the heatmap analysis of the top 100 most variable proteins (Fig. 2B). The majority of proteins quantified were cytoplasmic (Fig. 2C), and the most common molecular function identified was enzymatic (Fig. 2D). Cellular compartment and molecular subtype were conserved across the six brain regions with average RSD of 3.3% and 5.9%, respectively (Fig. 2C, D). Approximately 57% of the proteins (*n* = 3506) were quantified in all six brain regions (Supplementary Fig. 1).

### Regional differential abundance of proteins in AUD compared to control groups

**Fig. 3:**
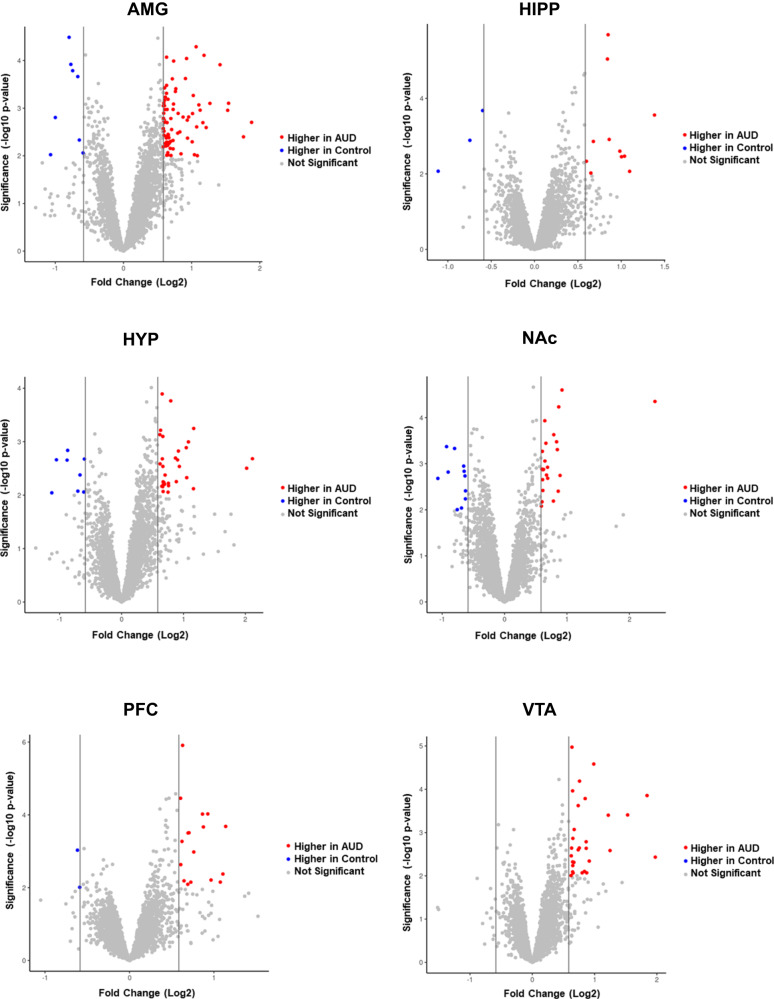
Volcano plot of proteins with differential abundance between AUD and control. Differentially expressed proteins (FC cutoff = 1.5, *p* < 0.01) between AUD and control groups in amygdala (AMG), hippocampus (HIPP), hypothalamus (HYP), nucleus accumbens (NAc), prefrontal cortex (PFC) and ventral tegmental area (VTA).

**Fig. 4:**
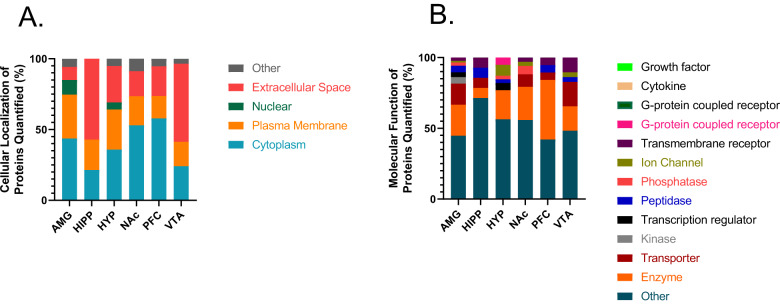
Cellular compartment and molecular functions of differentially expressed proteins between AUD and control groups. Cellular compartment (**A**) and molecular function (**B**) of proteins with differential abundance between AUD and controls (FC cutoff = 1.5, *p* < 0.01) from all brain regions.

The number of proteins with significant differential abundance (FC cutoff ± 1.5, *p* < 0.01) between AUD and control groups was quantified for each of the six brain regions (Table 1, Fig. 2A–F, Supplementary Tables 3–8). In all brain regions, there were more proteins elevated in AUD individuals than in control individuals (Fig. 3). Unique and common proteins identified with altered abundance across the six brain regions are shown in Supplementary Fig. 2. The AMG had the highest number of proteins (*n* = 87) followed by the HYP (*n* = 39), NAc (*n* = 32), VTA (*n* = 29), PFC (*n* = 19) and HIPP (*n* = 14). These proteins were largely localized to the cytoplasm, plasma membrane and extracellular space in all six brain regions (Fig. 4A), with nuclear protein localization observed in the AMG and HYP only. Extracellular space localized proteins were observed more in the HIPP and VTA regions compared to others (Fisher’s exact test, *p* < 0.05 and *p* < 0.01, respectively). (Fig. 4A). Molecular functions of the differentially abundant proteins were predominantly G-protein coupled receptors in addition to enzyme, transporter, kinase, and ion channel proteins (Fig. 4B). In the AMG and VTA, transporters such as solute carrier family proteins were among the altered proteins (Fig. 4B, Supplementary Tables 3 and 5), while in the HYP, ion channel proteins including GRIN1 (glutamate ionotropic receptor NMDA type subunit 1) were found to be elevated in AUD patients (Fig. 4B).

### Pathway alterations in AUD individuals by brain region

**Fig. 5:**
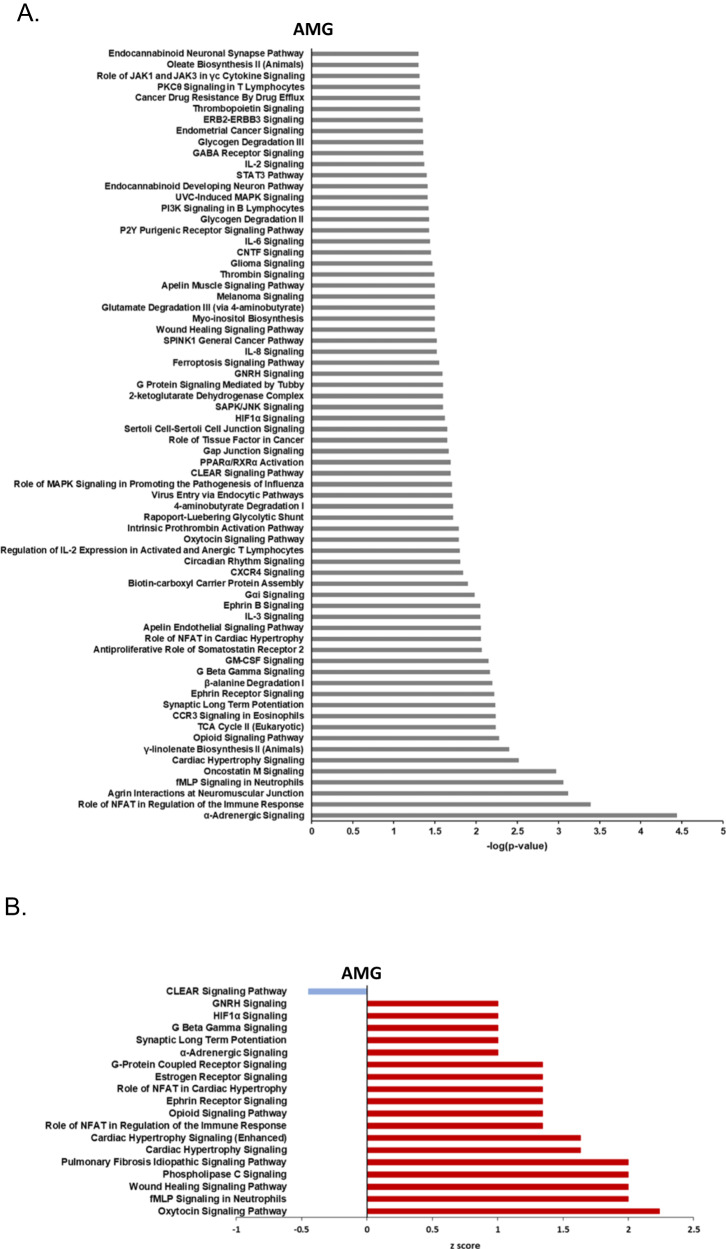
Canonical pathway analysis of differentially expressed proteins between AUD and control groups in AMG. Pathways impacted by AUD in **A** amygdala (AMG). **B** activated or inactivated pathways in AMG impacted by AUD. Ingenuity Pathway Analysis showing activated (red bars; positive *z*-scores) and inactivated pathways (blue bars; negative *z*-score).

**Fig. 6:**
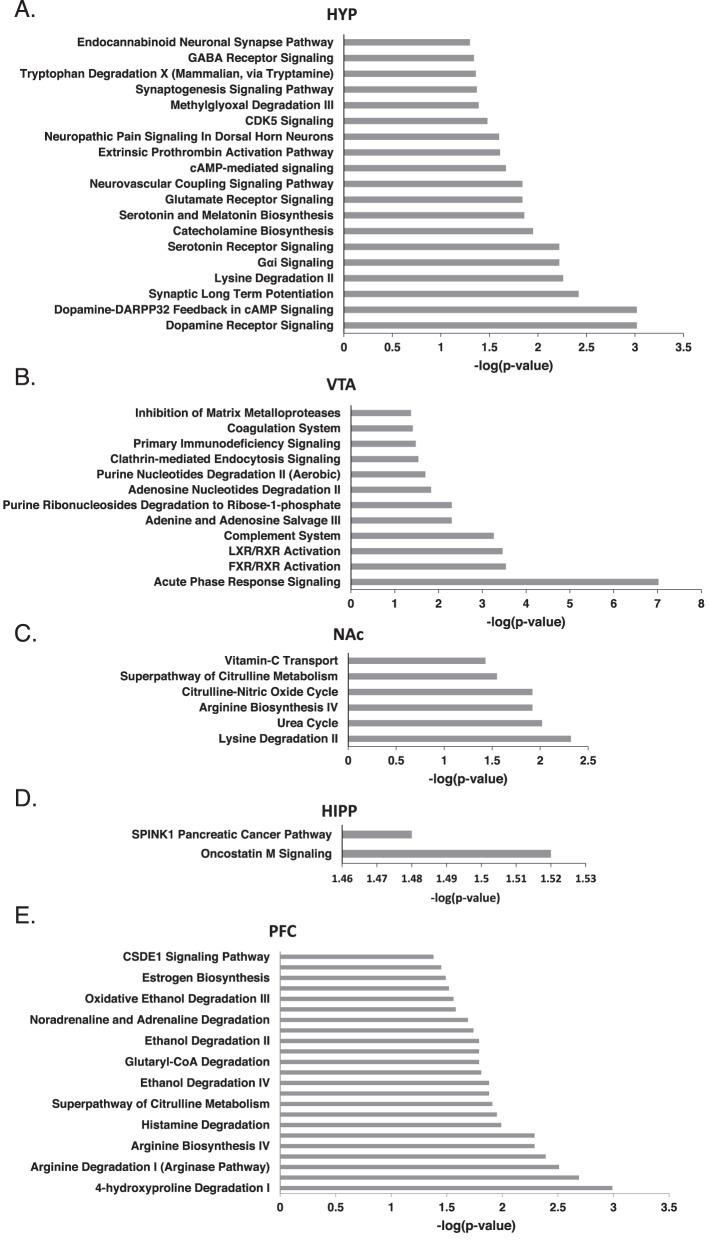
Canonical pathway analysis of differentially expressed proteins between AUD and control groups in brain regions. Pathways impacted by AUD in **A** hypothalamus (HYP), **B** ventral tegmental area (VTA), **C** nucleus accumbens (NAc), **D** hippocampus (HIPP) and **E** prefrontal cortex (PFC).

AUD was associated with diverse pathway alterations in each of the brain regions (Figs. 5 and 6). Pathway analysis was performed using significantly altered proteins (FC cutoff ± 1.5, *p* < 0.01) quantified between AUD and control groups (Table 1, Supplementary Tables 9–14). The AMG had the greatest number of enriched, AUD-impacted pathways among all brain regions analyzed. (Fig. 5A, Supplementary Table 9). Among these, α-Adrenergic Signaling was the most significant (Fig. 5A, Supplementary Table 9), and GABA Receptor Signaling was also enriched in AUD in AMG (Fig. 4A). In addition, there were 13 activated pathways and 1 inhibited pathway that were predicted by IPA (Fig. 5B). The AMG was the only brain region that had pathways passing the *z*-score cutoff ≤ |2|. The oxytocin signaling pathway was significantly enriched (Fig. 5A) and predicted to be activated in the AUD AMG where five proteins (GNB1, HRAS, HSPB1, PPP3CC, RRAS2) were elevated in AUD (Fig. 5B, Supplementary Table 9). Other activated pathways in the AMG included Opioid Signaling, Ephrin Receptor Signaling and pathways involved in wound healing and immune response (Fig. 5B, Supplementary Table 9). The CLEAR (Coordinated Lysosomal Expression and Regulation) pathway, which is associated with lysosomal/autophagy and may possibly play a role in the pathogenesis of neurodegenerative diseases, was predicted to be inactivated in the AMG (Fig. 5B, Supplementary Table 9).

In the HYP, dopamine receptor signaling and Dopamin-DARPP32 Feedback in cAMP Signaling were enriched where three (ADCY1, DDC, PPP1R1B) and four proteins (ADCY1, GRIN1, KCNJ15, PPP1R1B) were identified to be associated with these two pathways respectively (Fig. 6A, Supplementary Table 10). ADCY1, DDC and GRIN1 were greater, while KCNJ16 and PPP1R1B were lower in abundance (Supplementary Table 4). Other receptor signaling pathways enriched in the HYP impacted by AUD include Serotonin Receptor, Glutamate Receptor and GABA Receptor (Fig. 6A).

In the VTA, Acute Phase Response Signaling was enriched with six proteins (A2M, APOA1, C1QB, SERPINA3, SERPIN1, TF) associated with the pathway being elevated in AUD (Fig. 6B, Supplementary Table 11). In the NAc, pathways such as Lysine Degradation and Urea Cycle were enriched (Fig. 6C, Supplementary Table 12). In the PFC, ALDH4A1, elevated in the AUD, is associated with several degradation pathways, including 4-Hydroxyproline and Proline Degradation, Ethanol Degradation, Dopamine Degradation and Serotonin Degradation (Fig. 6F, Supplementary Tables 8 and 14).

Many of the pathways altered by AUD were unique to each of the brain regions assessed. Several pathways were enriched in both AMG and HYP, including Synaptic Long-Term Potentiation, Gαi Signaling, GABA Receptor Signaling and Endocannabinoid Neuronal Synapse Pathway. (Supplementary Fig. 3). Oncostatin M Signaling, which is associated with immune response, was enriched in the AMG and HIPP with elevated HRAS, RRAS2 in the AMG and elevated CHI3L1 in both brain regions (Fig. 5E, Supplementary Fig. 3, Supplementary Tables 9 and 13).

## Discussion

We report here the effect of AUD on protein abundance in six brain regions that are associated with the development and maintenance of addiction to alcohol. Greater than 6000 proteins, predominantly cytoplasmic enzymes, were quantified in total and the results clustered by brain region rather than by subject group. PCA analysis revealed three separate clusters, one comprised of only proteins in VTA and HYP, with HIPP, PFC and NAc forming another cluster that overlapped with AMG proteins. This raises the question of whether anatomic (similar cell types) and/or functional connectivity underlies these regional clusters.

The brain region with the greatest number of differentially expressed proteins was the AMG which is central to neurocircuitry, underlying stress, emotionality, and negative reinforcement, all features of severe AUD [2]. There is translational support for this finding, as reported in a recent study by Augier and colleagues [26], who developed an animal model with traits that map onto the diagnostic criteria of severe AUD; they examined gene expression across similar brain regions (NAc, PFC, HIPP, AMG) and found that the AMG had the highest number of significant alterations.

IPA indicated that AUD was associated with an enrichment of proteins in the α-adrenergic signaling pathway in the AMG (Fig. 5A; Supplementary Table 9). Adrenergic neurons project to the AMG and are implicated in the sensitization of stress systems that occur with chronic, compulsive drinking [27, 28]. Notably, pharmacologic agents that reduce adrenergic signaling, e.g., prazosin, doxazosin and propranolol, have been shown to reduce drinking in animal [28] and human studies [27].

The opioid signaling pathway was also enriched in the AMG and predicted to be activated (Fig. 5A, B; Supplementary Table 9). Mu opiate receptors are highly expressed [29] in the AMG and modulate the positive rewarding effects of alcohol [30]. Indeed, the mu opioid antagonist, naltrexone, is FDA-approved for the treatment of OUD. In addition, kappa-opioid receptors (KORs), expressed in the AMG, are elevated in animal models of AUD and are thought to play a role in negative reinforcement [31]. Nalmefene, a mu opiate receptor inverse agonist and kappa receptor antagonist, is approved in Europe for the treatment of AUD. The differentially abundant proteins associated with this pathway partially overlap with altered proteins in the oxytocin and alpha-adrenergic pathways.

In the AMG, the oxytocin signaling pathway was enriched and predicted to be activated in AUD (Fig. 5A, B; Supplementary Table 9). Chronic AUD is associated with the loss of oxytocin immunoreactivity in the paraventricular nucleus of the hypothalamus [32]. In alcohol-dependent rats, there is a decreased level of oxytocin in hypothalamic nuclei with elevation of oxytocin receptors in frontal and striatal brain regions [33]. In a human postmortem study of AUD subjects (using the same cohort as the current study), we reported an upregulation of oxytocin mRNA in the PFC of the AUD group compared to controls [21]. This may represent a compensatory upregulation of hypothalamic or extra-hypothalamic oxytocin synthesis [34]. The effect of exogenous oxytocin to reduce drinking behavior in rodents is dependent on oxytocin receptor signaling in the extended AMG [34]. Overall, the present results build evidence supporting the role of oxytocin in AUD and its potential as a pharmacotherapeutic target [35], though the mechanism for this potential therapeutic effect is unknown.

Glutamatergic signaling is elevated with chronic alcohol use. There is increased glutamatergic transmission and hyper-excitability during withdrawal, abstinence and chronic alcohol consumption [36]. The vesicular glutamate transporter VGLUT1 (SLC17A7) was increased in AUD compared to control subjects and is widely expressed in mesolimbic regions, including the AMG. VGLUT1 mRNA was increased 5-fold after binge drinking in the dorsal raphe nucleus [37]: longstanding increases in VGLUT1 mRNA and protein levels have also been reported after methamphetamine exposure in the striatum [38].

Protein kinase CK1 delta (CSNK1D) was significantly elevated in the AMG of AUD subjects (*p* < 0.01, FC ± 1.5). It is a ubiquitous serine/threonine kinase, regulates multiple cellular processes and is induced in the context of stress. It was increased in enriched pathways associated with immune response and gap junction signaling pathways (Supplementary Table 9). After chronic ethanol exposure, mRNA expression of CSNK1D in PFC was significantly positively correlated with ethanol consumption in rats [39]. We report a related isoform, protein kinase CK1 epsilon (CSNK1E), was significantly reduced in the NAc of AUD subjects. The same finding was reported in a rodent model of alcohol dependence, where this isoform was significantly reduced in alcohol-preferring rats in the NAc [39].

Ras-related protein (RRAS2), G Protein Subunit Alpha 14 (GNA14) and Protein Phosphatase 3 Catalytic Subunit Gamma (PPP3CC) are enriched in the AMG in AUD across numerous canonical pathways listed in Supplementary Table 9. RRAS2 functions as a GTPase is located on the plasma membrane and is involved in signal transduction. In alcohol-preferring rats, gene expression was reduced in the VTA [40]. GNA14 and PPP3CC have not yet been studied in the context of the effect of alcohol on their expression [41].

There was an increase in the abundance of gamma-aminobutyric acid type A receptor subunit Alpha2 (GABRA2) in the AMG of individuals with AUD compared to controls. Chronic alcohol exposure results in increased GABA-ergic tone in the AMG [42], and intra-AMG infusion of a GABA-A receptor agonist suppresses drinking in alcohol-dependent rats [43], but it is unclear what role the increased abundance of subunit alpha2 plays, if any, in this adaptation to chronic alcohol exposure.

Canonical pathways enriched in AUD subjects in the HYP (Supplementary Table 10) were related to neurotransmitter signaling (dopamine, serotonin, glutamate, GABA, G protein, endocannabinoid), neuroplasticity (synaptic long-term potentiation, CDK5 signaling, synaptogenesis signaling) and biosynthesis (catecholamine, serotonin/melatonin) and degradation (methylglyoxal, tryptophan). Methylglyoxal is a metabolic byproduct of alcohol metabolism, so it is not surprising that we find its degradation pathway elevated in AUD. Preclinical studies report that alcohol consumption results in the upregulation of dopamine transporter (DAT) [44] and increased serotonin release [45] in the lateral hypothalamus (LH). Alcohol-dependent rats have significantly increased hypothalamic glutamate transporter EAAC-1 and GABA transporter [46]. The LH has reciprocal neural connections with the VTA, which are composed of both glutamatergic and GABA-ergic projections, which mediate avoidant and reward-seeking behaviors, respectively [47]. The LH also receives projections from the extended AMG, which, in late-stage addiction, undergoes upregulation of stress neurotransmitters [48]. Of note, oxytocin neurons project from the supraoptic and paraventricular nucleus of the HYP to the AMG, where oxytocin signaling was elevated (see above). Hypothalamic glutamatergic inputs to magnocellular oxytocin neurons in the PVN and SON facilitated synchronous firing of oxytocin neurons [49], raising the possibility that the elevated glutamate signaling in the HYP may be contributing to upregulation of oxytocin signaling in the AMG.

In the AMG and, to some degree, VTA, in AUD, immune pathways were enriched, such as pro-inflammatory IL-3, IL-8, IL-2, IL-6 Signaling and PPARα/RXRα Activation in AMG (Supplementary Table 9) as well as Acute Phase Response Signaling and Complement System activation in VTA (Supplementary Table 11). Chronic, heavy alcohol use leads to ethanol-induced neuroimmune activation characterized by activation of toll-like receptors, which alter neural function and can, in turn, impact alcohol consumption behaviors [50]. One mechanism of TLR4 activation is via inhibition of histone deacetylases (HDACs) by ethanol [51]. Consistent with this observation, we found that HDAC11 was significantly lower in abundance in AMG in individuals with AUD compared to controls. As such, immune modulators are being investigated for treating AUD, such as ibudilast, peroxisome proliferator-activated receptor (PPAR) agonists, minocycline, phosphodiesterase 4 (PDE-4) inhibitor, and N-acetylcysteine [52]. Tenascin C was significantly reduced in AUD subjects across four of the brain regions (AMG, PFC, NAc, and HIPP), especially in PFC and NAc. Lastly, Tenascin C is an extracellular matrix protein and a pro-inflammatory mediator that activates TLR4 [53]; however, little is known about the effect of chronic alcohol exposure on Tenascin C function.

Neurogranin (Ng) modulates NMDAR-mediated Ca^+2^ calmodulin signaling. Ng was reduced in HYP, NAc and HIPP in AUD subjects compared to controls. Ng null mice self-administer significantly more alcohol and display reduced aversive motivation [54]. NAc Ng regulates NMDAR and mGluR5 signaling and may play a role in altering aversive motivation for alcohol [54].

The enriched pathways identified in this comprehensive proteomic analysis of six brain regions in subjects with AUD yielded a list of potential therapeutic targets for AUD. Notably, targets that emerged here from significantly enriched pathways, are being investigated as therapeutic targets for AUD, such as oxytocin receptor, GABAB receptor, α-adrenergic receptors, cannabinoid receptor, opioid receptor, and PPAR. Additional protein targets identified from highly differentially expressed proteins (*p* < 0.01, FC cutoff = 1.5) using IPA include ABAT, CSNK1D, PPP3CC, GABBR2 and SLC1A3 (Supplementary Tables 3–8). A selective CSNK1D and CSNK1E inhibitor, PF-670432, prevents relapse-like alcohol drinking in rats [55]. Cyclosporin A that targets PPP3CC decreases binge-like drinking in mice [56]. The selective GABA-B receptor agonist baclofen has been investigated in AUD and reduces drinking in individuals with AUD and alcohol-associated liver disease [57]. Lastly, Riluzole that targets SLC1A3 reduces ethanol self-administration and ethanol withdrawal symptoms in mice [58].

This study had several limitations. The cohort was comprised entirely of males, so generalization of these results to biological variables related to AUD in females was not possible. All the subjects were smokers, and all but one of the control subjects was a nonsmoker. Therefore, it is not possible to disentangle the effects of chronic alcohol from nicotine/smoking exposure.

Overall, this study provides a hypothesis-generating proteomic analysis examining the effects of AUD in six brain regions involved in the pathogenesis of AUD. The AMG contained the greatest number of differentially abundant proteins and altered molecular pathways. While there are altered pathways identified across several of the regions analyzed, the large number of altered pathways unique to each brain region highlights the neurobiological complexity of this disease, which merits further investigation.

### Supplementary information

Supplementary Figure and Table Legends

Supplementary Fig 1-3

Supplementary Table 1

Supplementary Table 2

Supplementary Table 3

Supplementary Table 4

Supplementary Table 5

Supplementary Table 6

Supplementary Table 7

Supplementary Table 8

Supplementary Table 9

Supplementary Table 10

Supplementary Table 11

Supplementary Table 12

Supplementary Table 13

Supplementary Table 14

## Data Availability

Protein abundance between the AUD and control groups data across all six brain regions are publicly available at www.lmdomics.org/AUDBrainProteomeAtlas/. The mass spectrometry proteomics data have been deposited to the ProteomeXchange Consortium (http://proteomecentral.proteomexchange.org) via the PRIDE [59] partner repository with the dataset identifier PXD040884.
