# Supplementary material for: Brain proteomic atlas of alcohol use disorder in adult males
Source: Transl Psychiatry. 2023 Oct 13;13:318. doi: 10.1038/s41398-023-02605-0 (PMC10575941; doi:10.1038/s41398-023-02605-0)
Supplement: Supplementary file 1 — Supplementary Figure and Table Legends [file 41398_2023_2605_MOESM1_ESM.docx]

**Supplementary Materials**

**Supplementary Figures**

**Supplementary Figure 1. Upset plot of number of total proteins in each brain region.** Abbreviations: Amygdala (AMG), hippocampus (HIPP), hypothalamus (HYP), nucleus accumbens (NAc), prefrontal cortex (PFC), ventral tegmental area (VTA).

**Supplementary Figure 2. Upset plot of number of proteins with differential expression between AUD and control (FC cutoff > 1.5, p < 0.01).** Abbreviations: Amygdala (AMG), hippocampus (HIPP), hypothalamus (HYP), nucleus accumbens (NAc), prefrontal cortex (PFC), ventral tegmental area (VTA).

**Supplementary Figure 3. Heatmap of common enriched pathways among brain regions.** Abbreviations: Amygdala (AMG), hippocampus (HIPP), hypothalamus (HYP), nucleus accumbens (NAc), prefrontal cortex (PFC). Pathways not enriched in the brain region is represented by white colored blocks.

**Supplementary Tables**

**Supplementary Table 1. Patient cohort.**

**Supplementary Table 2. Proteins identified from each brain region.**

**Supplementary Table 3 - 8. Proteins with differential expression (FC cutoff = 1.5, p < 0.1) between AUD and control from amygdala, hypothalamus, VTA, nucleus accumbens, hippocampus and prefrontal cortex.**

**Supplementary Table 9 - 14. Pathways from proteins with differential expression (FC cutoff = 1.5, p < 0.1) between AUD and control from amygdala, hypothalamus, VTA, nucleus accumbens, hippocampus and prefrontal cortex.**
