# Supplementary material for: Brain proteomic atlas of alcohol use disorder in adult males
Source: Transl Psychiatry. 2023 Oct 13;13:318. doi: 10.1038/s41398-023-02605-0 (PMC10575941; doi:10.1038/s41398-023-02605-0)
Supplement: Supplementary file 2 — Supplementary Fig 1-3 [file 41398_2023_2605_MOESM2_ESM.pptx]

## Slide 1
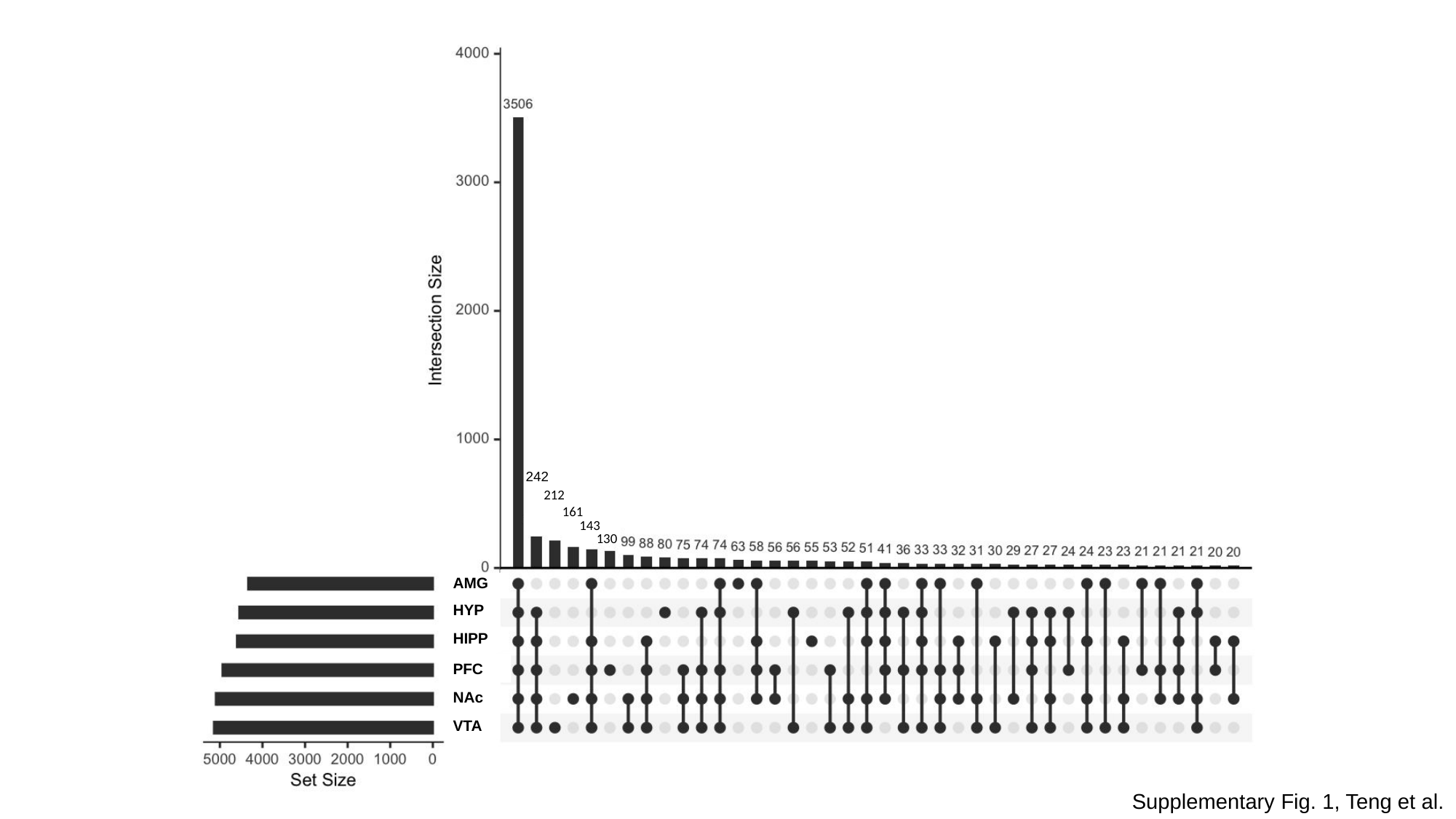

AMG
HYP
HIPP
PFC
NAc
VTA
242
212
161
143
130
Supplementary Fig. 1, Teng et al.

## Slide 2
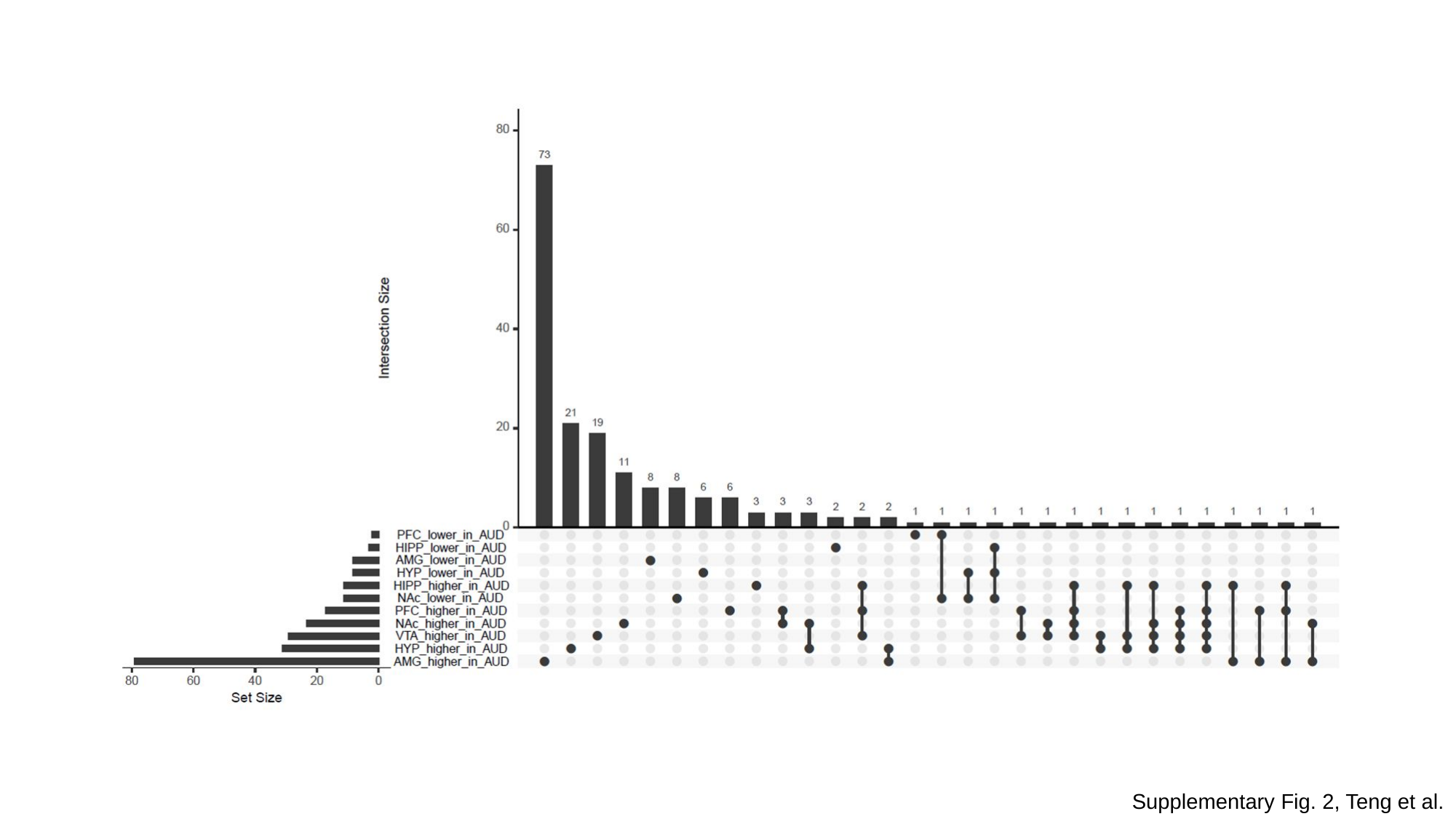

Supplementary Fig. 2, Teng et al.

## Slide 3
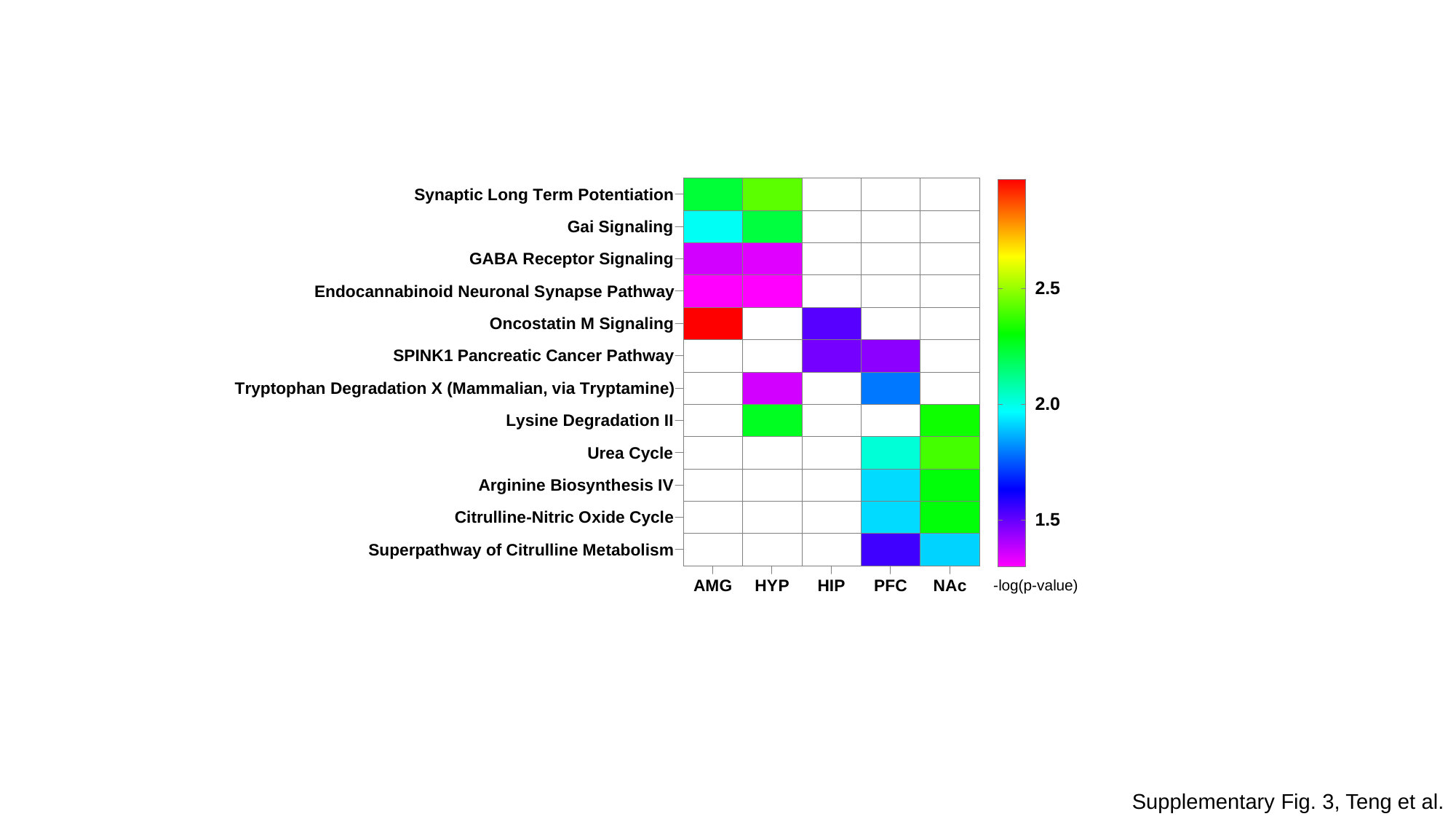

-log(p-value)
Supplementary Fig. 3, Teng et al.
